# IgG-Binding Peptidomimetic Mixed-Charge Polymer-Modified Resins for Chromatographic Purification of Antibodies

**DOI:** 10.1021/acsami.4c16861

**Published:** 2024-11-26

**Authors:** Koichi Deura, Akihiro Sakama, Yasuhiro Moriwaki, Daniel Citterio, Yuki Hiruta

**Affiliations:** † Department of Applied Chemistry, Faculty of Science and Technology, Keio University, 3-14-1 Hiyoshi, Kohoku-ku, Yokohama, Kanagawa 223-8522, Japan; ‡ Division of Basic Biological Sciences, Faculty of Pharmacy, Keio University, 1-5-30 Shibakoen, Minato-ku, Tokyo 105-8512, Japan

**Keywords:** antibody purification, peptidomimetics, pH-responsive polymer, mixed-charge polymer, chromatography

## Abstract

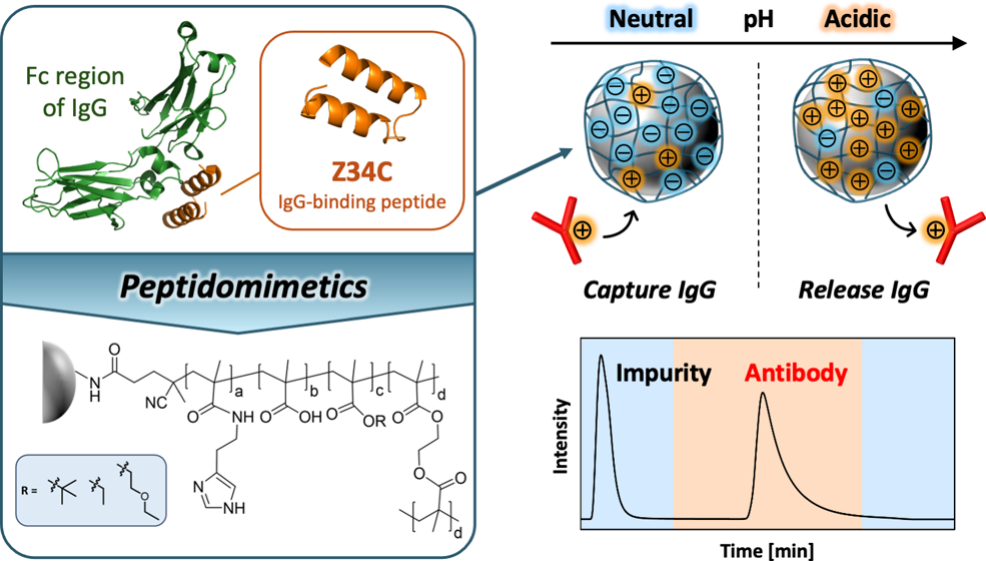

The process of antibody purification using Fc affinity ligands such as protein A, G, and L faces several challenges including high cost, low stability, and loss of antibody activity due to harsh elution conditions. Here, we describe a chromatographic purification of antibodies utilizing a pH-responsive mixed-charge polymer that mimics the IgG-binding peptide (Z34C) derived from the B domain of protein A. The protein A mimetic resins were prepared by modifying the surface of a TOYOPEARL, methacrylate resin with a polymer that mimics the amino acid sequence of Z34C and the functions of histidine and acidic and neutral amino acids using histamine methacrylamide (HisMA), methacrylic acid, and neutral monomers. The therapeutic monoclonal antibody (mAb), rituximab, was retained on the column at pH 7 and eluted under mildly acidic conditions at pH 5 using a protein A mimetic resin (HisMA20-EEMA) optimized for antibody interaction. The injected antibodies were selectively captured on the column by hydrophobic and electrostatic interactions with the protein A mimetic polymer under neutral conditions and eluted by electrostatic repulsion under acidic conditions. The HisMA20-EEMA column successfully purified mAbs from mixtures with BSA, mouse ascites fluid, and hybridoma cell culture supernatant. In addition, the HisMA20-EEMA column consistently achieved 90% antibody recovery in 100 consecutive purifications from cell culture supernatant. The antibody purification method presented in this study is low cost, highly durable, easy to synthesize, and allows for mild elution conditions. The results demonstrate that the approach of mimicking IgG-binding peptides with mixed-charge polymers is useful for the development of column packing materials for antibody purification.

## Introduction

With the rapid increase in demand for therapeutic monoclonal antibodies (mAbs) in recent years, improvements in downstream purification processes have been actively investigated to reduce manufacturing costs.^1−3^ Currently, the typical purification process is affinity chromatography using protein A, G, and L as affinity ligands.^4,5^ However, these processes face several challenges that are difficult to overcome, including high cost, low stability, harsh elution conditions, and potential ligand leakage.^6−8^ To address these issues, mAb-binding peptide-based affinity capture and recovery of mAbs have been studied.^9−14^ Although peptide ligands have achieved selective isolation of mAbs, complex ligand synthesis and the difficulty in antibody release from the adsorbents due to excessive affinity remain challenges.

Therefore, the development of low-cost and easy-to-synthesize small molecule ligands has been promoted.^15−18^ One of the main examples is hydrophobic charge-induced chromatography (HCIC) using 4-mercaptoethylpyridine, in which antibodies captured by hydrophobic interactions at neutral pH are eluted by electrostatic repulsion between the positively charged ligand and the antibody at acidic pH.^15^ However, nonspecific binding to foreign substances has been a problem.^19^ Histidine ligand affinity chromatography (HLAC), a type of pseudobiospecific chromatography, is an antibody purification method with the advantages of low cost and nontoxicity.^20^ In HLAC, the imidazole ring has been shown to bind to IgG mainly through hydrogen-bonding, van der Waals, and hydrophobic interactions,^20^ and efficient antibody purification with polymer materials based on 1-vinylimidazole, a simple nitrogen heterocyclic molecule, has been reported.^21^

Mixed-mode ligands, which typically contain hydrophobic and ionic moieties, exhibit excellent performance in separating antibodies from complex samples.^22−25^ By optimizing the type and composition of functional monomers, synthetic polymers with affinity for target molecules through multiple interactions can be fabricated. Shea and co-workers reported a method for preparing synthetic polymer hydrogel nanoparticles with high affinity for IgG by optimizing the composition of functional monomers using an iterative process.^26^ Stimuli-responsive polymers can drastically switch their affinity for target compounds and proteins in response to external stimuli.^27^ Nagase and co-workers successfully purified an antibody by changing the column temperature using a temperature-responsive anionic polymer with optimized electrostatic and hydrophobic interactions with the antibody.^28,29^ We have previously developed an antibody separation system using a thermoresponsive polymer column by changing buffer ion species at constant temperature.^30^ While this technique is inexpensive and avoids elution under conditions that damage antibodies, there were concerns for industrialization, such as uniform temperature control in large scale columns.

In recent years, the functions of relatively simple peptides have been reproduced in synthetic polymers using monomers that mimic the structures of key amino acids. Due to their stability and versatility, peptidomimetic polymers have attracted attention as a means of developing functional materials with diverse properties at low cost.^31−35^ Here we propose an approach to mimic the function of IgG-binding peptides using pH-responsive mixed-charge polymers. The B domain of protein A, which binds specifically to the Fc region of IgG, consists of a bundle of three α-helices,^36^ and its interaction with IgG involves mainly hydrophobic interactions and a few polar or electrostatic interactions.^37^ We have previously developed a chromatographic system by modifying the surface of column packing material with pH-responsive mixed-charge polymers that switches between cation and anion exchange modes in response to changes in the mobile phase pH.^38^ The surface charge of the pH-responsive mixed-charge polymer switches in response to the external pH change, and the charge balance can be adjusted by changing the ratio of cationic to anionic monomers.^39,40^ In this study, we applied this technology to mimic the interaction of protein A and the Fc region of IgG by using pH-responsive mixed-charge polymers that mimic the amino acid sequence of the IgG-binding peptide Z34C^41^ derived from the B domain of protein A. This includes cationic monomers that mimic the structure of histidine, which is important for interaction with antibodies.^42^ The protein A mimetic mixed-charge polymer showed high affinity for antibodies and allowed switching of antibody adsorption and desorption by changing the mobile phase pH from neutral to acidic, similar to the function of protein A. This simple procedure to purify mAbs from mixtures containing typical contaminants was achieved using a column packed with protein A mimetic polymer-modified resins. In addition, the durability and reusability of the packing material were investigated.

## Experimental Section

### Materials

Histamine dihydrochloride, methacryloyl chloride, methacrylic acid (MAA), *tert*-butyl methacrylate (*t*BMA), ethyl methacrylate (EMA), and 2-ethoxyethyl methacrylate (EEMA) were purchased from Tokyo Chemical Industries (Tokyo, Japan). MAA, *t*BMA, EMA, and EEMA were purified by distillation under reduced pressure before use. Di-*tert*-butyl dicarbonate (Boc_2_O), acetyl chloride (AcCl), 4,4′-azobis(4-cyanovaleric acid) (V-501), ethylene glycol dimethacrylate (EGDMA), and bovine serum albumin (BSA, globulin-free) were purchased from FUJIFILM Wako Pure Chemical Corporation (Osaka, Japan). 2-Ethoxy-1-ethoxycarbonyl-1,2-dihydroquinoline (EEDQ) and mouse ascites fluid were purchased from Sigma-Aldrich (St. Louis, MO). Triethylamine (Et_3_N) and ninhydrin were purchased from Nacalai Tesque (Kyoto, Japan). TOYOPEARL AF-Amino-650 M was purchased from Tosoh Corporation (Tokyo, Japan). Rituximab was purchased from Zenyaku Kogyo (Tokyo, Japan) and Kyowa Kirin (Tokyo, Japan). Trastuzumab was purchased from Daiichi Sankyo Corporation (Tokyo, Japan). A PURELAB water purification system (ELGA Veolia Water, Marlow, U.K.) was used to obtain the ultrapure water used for the preparation of all aqueous solutions. Phosphate buffer (PB) was prepared from NaH_2_PO_4_/Na_2_HPO_4_, and citric acid buffer (CAB) was prepared from citric acid/trisodium citrate. ^1^H NMR spectroscopy was performed using a JNM-ECS400 or JNM-ECA500 spectrometer (JEOL, Tokyo, Japan) at 298 K.

### Synthesis of HisMA

Synthesis of HisMA was carried out according to a previously reported work with modification (Scheme S1).^43^ Boc-HisMA was obtained through Schotten–Baumann reaction of histamine dihydrochloride and methacryloyl chloride followed by the protection of the imidazole nitrogen with *tert*-butoxycarbonyl (Boc) group for purification. Then Boc-HisMA was deprotected to obtain HisMA as HCl salt. The detailed synthetic procedures are shown in the Supporting Information.

### General Procedure for Synthesis of Protein A Mimetic pH-Responsive Mixed-Charge Polymer-Modified Resins

Initiator-modified TOYOPEARL resin and polymer-modified TOYOPEARL resin were prepared according to previously reported works with modification by changing the resin and monomers.^28,38^ First, polymerization initiator V-501 was immobilized on TOYOPEARL AF-Amino-650 M resin (particle size: 65 μm, pore size: 100 nm) by reacting with amino groups on the surface of the resins (Scheme S2) as follows. V-501 (1.17 g, 3.75 mmol) and EEDQ (2.06 g, 7.50 mmol) were dissolved in DMF (30 mL) in a 100 mL round-bottom flask. Then, TOYOPEARL resin (2.00 g) was added to the solution. The mixture was degassed with N_2_ bubbling for 30 min and stirred at room temperature for 22 h. V-501-modified resin was filtered, rinsed with ethanol (150 mL), and dried under reduced pressure. Then, a pH-responsive mixed-charge polymer consisting of pH-responsive cationic, pH-responsive anionic, and neutral monomers and cross-linkers was modified on the surface of the TOYOPEARL resin by random copolymerization. HisMA·HCl as pH-responsive cationic monomer, MAA as pH-responsive anionic monomer, the corresponding neutral monomer, and EGDMA (2 mol %) as cross-linker were dissolved in methanol (30 mL) in a 100 mL round-bottom flask. V-501 modified TOYOPEARL resin of the same weight as the total monomer weight was added to the solution. The mixture was degassed by N_2_ bubbling for 30 min and stirred at 70 °C for 5 h. Polymer-modified resin was filtered, rinsed with methanol (100 mL), and dried under reduced pressure.

#### HisMA20-*t*BMA

HisMA·HCl (78 mg, 0.36 mmol), MAA (31 mg, 0.36 mmol), *t*BMA (149 mg, 1.05 mmol), and EGDMA (7.1 mg, 0.036 mmol) were dissolved in methanol (30 mL).

#### HisMA20-EMA

HisMA·HCl (42 mg, 0.20 mmol), MAA (17 mg, 0.20 mmol), EMA (65 mg, 0.57 mmol), and EGDMA (3.9 mg, 0.020 mmol) were dissolved in methanol (30 mL).

#### HisMA20-EEMA

HisMA·HCl (52 mg, 0.24 mmol), MAA (21 mg, 0.24 mmol), EEMA (111 mg, 0.700 mmol), and EGDMA (4.8 mg, 0.024 mmol) were dissolved in methanol (30 mL).

### Characterization of Protein A Mimetic Mixed-Charge Polymer-Modified Resins

The degree of modification of V-501 to the surface of TOYOPEARL resin was indirectly determined by comparing the amount of primary amine residues on the resin surface before and after modification. The ninhydrin reaction was utilized to determine the degree of modification. Briefly, dried resin (2 mg) was added to ethanol (400 μL), allowed to swell for 10 min, and then 0.1 M aqueous ninhydrin (100 μL) was added. The mixture was heated at 95 °C for 10 min. After cooling to room temperature, the mixture was diluted with ethanol (1.5 mL) and centrifuged, and the absorbance of the supernatant was measured by using a UV-1800 spectrophotometer (SHIMADZU, Kyoto, Japan). The same mixture without resin was measured as blank. The degree of modification was calculated from the difference in absorbance at 570 nm before and after modification. The shape of TOYOPEARL and V-501 or polymer-modified resins were observed by a scanning electron microscope (SEM, TM3030Plus, Hitachi, Tokyo, Japan). The prepared polymer-modified resins were characterized by CHN elemental analysis (UNICUBE; Elemental Analysensysteme GmbH, Langenselbold, Germany).

### Column Packing

Prepared pH-responsive mixed-charge polymer-modified resins were packed into a stainless-steel column (4.6 mm I.D. × 10 mm). Prepared resins were dispersed in methanol/chloroform (1:3 v/v). Then, the slurry was poured into a column packer connected to the column. Packing was performed by a HPLC pump (LC-20AR, SHIMADZU, Kyoto, Japan) under flow of methanol for 60 min, followed by methanol/water (50:50 v/v) for 30 min at a flow rate of 10 mL/min.

### Basic Characterization of Protein A Mimetic Columns

Retention and elution behavior of antibodies on protein A mimetic columns was studied using a Prominence-i LC2030C (SHIMADZU) system equipped with ultraviolet–visible (UV/vis) detector. All measurements were performed at a flow rate of 0.2 mL/min at 25 °C. 4 mg/mL rituximab (2 μL) was injected by an autosampler. The recovery rates of antibodies were calculated from the peak area of the chromatogram measured at 280 nm as 100% of the peak area of antibody samples (4 mg/mL, 2 μL) flowed with elution buffer (50 mM CAB at pH 3.0) without column. Protein A mimetic columns were equilibrated with eluent for at least 10 min before all measurements and washed with elution buffer (50 mM CAB at pH 3.0) for 5 min after measurements.

### Isocratic Elution of Antibodies

Following sample injection, the eluent flowed through the column for 5 min. Three different concentrations (10, 50, 100 mM) of PB at pH 7.0 as a binding buffer and three different pH (3.0, 4.0, 5.0) of 50 mM CAB as an elution buffer were investigated.

### Gradient Elution of Antibodies

Following sample injection, the column was washed for 3 min with the binding buffer (10 mM PB at pH 7.0). Three different pH (3.0, 4.0, 5.0) of 50 mM CAB were then applied as elution buffer for 6 min. Finally, the column was equilibrated for 11 min with the binding buffer.

### Antibody Purification from Mixtures with BSA, Mouse Ascites Fluid, and Hybridoma Cell Culture Supernatant

To investigate the effects of major contaminants introduced during the antibody production process, samples containing 2.0 mg/mL rituximab or trastuzumab were prepared with BSA, mouse ascites fluid, or hybridoma cell culture supernatant. Mixed samples were prepared by adding equal volumes of BSA solution (4 mg/mL), mouse ascites fluid, or hybridoma cell culture supernatant to antibody samples (4 mg/mL rituximab or trastuzumab), respectively. Cell culture supernatant was obtained by growing hybridoma cells^44^ for 3 days in RPMI 1640 medium containing 10% FBS. Each sample (10 μL) was injected on the HisMA20-EEMA column by the autosampler. For purification from cell culture supernatant containing rituximab at a concentration of 0.02 mg/mL, samples (1 mL) were injected into the column using an LC-20AR pump and a manual injector (Rheodyne 8125), and then the column was replaced with the LC2030C system for gradient elution. Following sample injection, the column was washed for 3 min with the binding buffer (10 mM PB at pH 7.0) to capture antibodies and wash away impurities. Subsequently, antibodies were eluted from the column by applying the elution buffer (50 mM CAB at pH 3.0 or 5.0) for 6 min. In each measurement, impurities eluting between 0.5 to 1.5 min were collected and defined as fraction 1. Rituximab eluted between 6.8 to 7.8/7.0 to 8.0 min or trastuzumab eluted between 6.7 to 7.7/6.6 to 7.6 min were collected and defined as fraction 2 in the measurement using pH 3.0/5.0 CAB as elution buffer.

### Determination of Purity of Purified Antibodies by Size Exclusion Chromatography (SEC)

A TSKgel guardcolumn UP-SW DC (4.6 mm I.D. × 2 cm) and a TSKgel UP-SW3000 column (4.6 mm I.D. × 15 cm) connected in a series were used to determine purity of purified antibodies eluted from the HisMA20-EEMA column. Eluted samples (fraction 1, fraction 2), samples before separation, mAbs (rituximab, trastuzumab), BSA, mouse ascites fluids, and hybridoma cell culture supernatant (20 μL for each sample) were analyzed on the SEC column. Each run was performed using a 10 min isocratic elution with PB (0.2 M, pH 6.7, 0.05% NaN_3_) at a flow rate of 0.35 mL/min at 25 °C.

## Results and Discussion

### Characterization of Protein A Mimetic Resins

**Figure 1:**
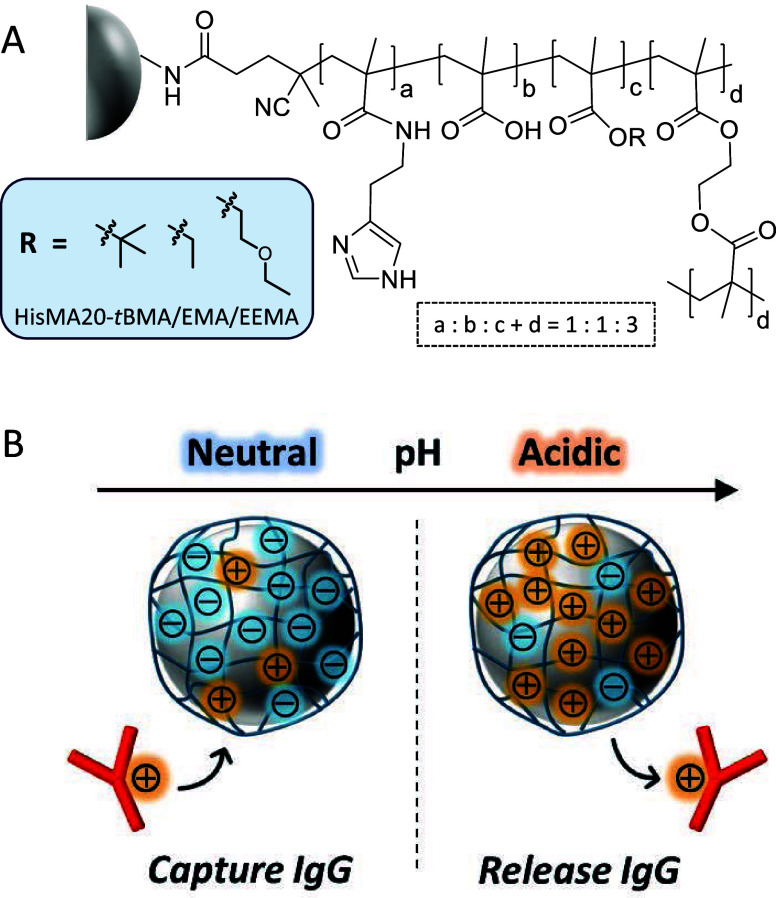
(A) Molecular structures of protein A mimetic pH-responsive mixed-charge polymer-modified TOYOPEARL resin. (B) Schematic illustration of pH-dependent interaction of IgG with the surface of TOYOPEARL resin modified with a protein A mimetic pH-responsive mixed-charge polymer.

Figure 1 shows a schematic illustration of the protein A mimetic pH-responsive mixed-charge polymer-modified resin. The ratio of cationic, anionic, and neutral monomers (hydrophobic monomer and cross-linker) in the polymer is 1:1:3, which mimics the amino acid sequence of Z34C,^41^ an IgG-binding peptide derived from protein A. In addition, HisMA with an imidazole unit was used as the cationic monomer to mimic the function of histidine. Imidazole in histidine contributes to the adsorption of protein A and the Fc region of IgG through hydrophobic interactions at neutral pH and their desorption through electrostatic repulsion due to its positive charge at acidic pH.^42^ Since carboxy groups have also been reported to be important for antibody binding via electrostatic interactions,^22^ MAA was used as the anionic monomer. To investigate the effect of different hydrophobicity of neutral monomers on the strength of antibody retention on the stationary phase, three protein A mimetic polymer-modified resins with different neutral monomers were prepared.

The ninhydrin reaction was utilized to determine the degree of modification of V-501 to the surface of TOYOPEARL resin. Ninhydrin is a reagent that reacts with primary amines to produce a purple color and is commonly used for the detection of amino acids and proteins.^45^ The carboxy group of V-501 reacted with amino groups on the surface of TOYOPEARL resin to form amide bonds. Therefore, the degree of modification can be calculated by comparing the number of primary amine moieties on the surface of resins before and after V-501 modification using ninhydrin. The results of absorbance measurement of ninhydrin reaction with the TOYOPEARL resin before and after V-501 modification are shown in Figure S1. Taking the absorbance at 570 nm of ninhydrin solution without resin and ninhydrin solution treated with TOYOPEARL resin as 0 and 100% amine content, respectively, it was found that 2.8% amine moiety was present on the surface of the V-501 grafted TOYOPEARL resin. The degree of modification was thus calculated to be 97.2%, confirming that V-501 was introduced into almost all amino groups on the TOYOPEARL resin surface. The protein A mimetic polymer-modified resins were characterized by CHN elemental analysis. Table 1 summarizes the elemental composition of TOYOPEARL, V-501 grafted TOYOPEARL, and polymer-modified TOYOPEARL resins. The V-501 grafted resins exhibited higher nitrogen content than the TOYOPEARL resins. This is due to the modification with V-501 having a higher content of nitrogen elements. The nitrogen content of polymer-modified resins was lower than that of V-501-modified resins, which is attributed to the successful modification of polymer containing nitrogen elements only in the imidazole moiety, resulting in the relatively lower contents of nitrogen elements. SEM images of the resins are shown in Figure S2. It was confirmed that the shape of the V-501 and polymer-modified resins (Figure S2B–E) was unchanged compared to that before modification (Figure S2A). These characterization results indicate that the resin surface has been successfully functionalized by polymer modification while maintaining the shape of the resin.

**Table 1:** Characterization of Protein A Mimetic Mixed-Charge Polymer Modification to TOYOPEARL Resins Utilizing CHN Elemental Analysis^a^

	elemental composition [%]		
	C	H	N
TOYOPEARL	51.66 ± 0.12	8.00 ± 0.02	0.76 ± 0.01
V-501-grafted TOYOPEARL	52.81 ± 0.07	7.74 ± 0.01	2.79 ± 0.01
HisMA20-*t*BMA	53.34 ± 0.24	7.88 ± 0.05	2.00 ± 0.01
HisMA20-EMA	52.70 ± 0.25	7.79 ± 0.03	2.02 ± 0.01
HisMA20-EEMA	52.72 ± 0.14	7.79 ± 0.01	2.03 ± 0.01

a Data represents mean values ± SD (*n* = 3).

### Antibody Retention and Elution Behavior of Protein A Mimetic Columns

**Figure 2:**
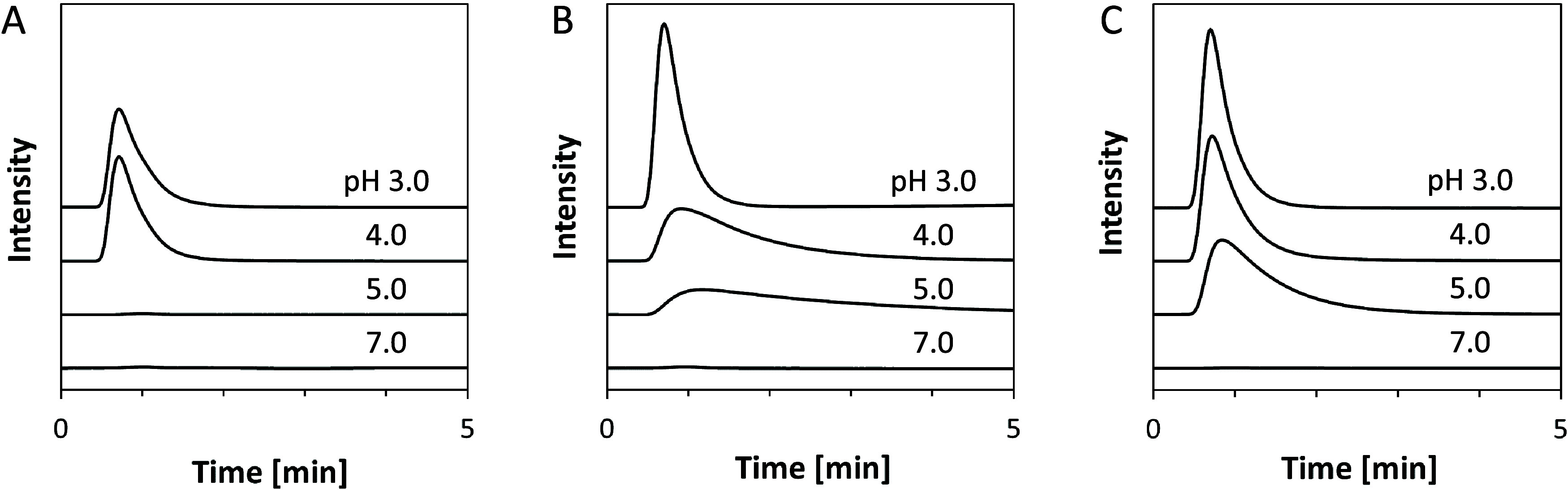
pH-dependent antibody retention and elution behavior of (A) HisMA20-*t*BMA (B) HisMA20-EMA (C) HisMA20- EEMA. Analytical conditions: mobile phase: 10 mM PB at pH 7.0, 50 mM CAB at pH 3.0/4.0/5.0; flow rate: 0.2 mL/min; analyte: 4 mg/mL rituximab; injection volume: 2 μL.

Prior to performing antibody purification using gradient elution, the basic antibody retention and elution performances of protein A mimetic columns were evaluated using buffers of various pH and salt concentrations. Figure 2 shows the pH-dependent retention and elution behavior of rituximab in three protein A mimetic columns with different hydrophobic monomers. All columns successfully retained rituximab using binding buffer (10 mM PB at pH 7.0). On the other hand, when acidic buffer was used as the mobile phase, the antibody was eluted from the columns. This pH-dependent retention and elution behavior of the antibody is similar to that of protein A columns, suggesting that the peptidomimetic polymer design successfully reproduced the function of protein A. The p*K*_a_ of PMAA and HisMA-based polymers were reported to be ∼5.5^46^ and 6.3,^47^ respectively. This predicts that the surface of the stationary phase is negatively charged under neutral conditions and that the positively charged rituximab (pI = 8.86^48^) is retained on the column through electrostatic and hydrophobic interactions. Here, like the histidine in the B domain of protein A, the imidazole of HisMA would have contributed to antibody binding through hydrophobic interactions at neutral pH, and its positive charge at acidic pH would have played a role in antibody elution by electrostatic repulsion. The lower the pH of the eluent, the easier it is for the antibody to elute, because the resin surface becomes more positively charged due to increased protonation. Notably, the HisMA20-EEMA column showed relatively sharp elution peaks even when using 50 mM CAB at pH 5.0 as eluent (Figure 2C), indicating that this column may be applicable to antibody purification under relatively mild acidic conditions.

Using three different concentrations (10, 50, 100 mM) of PB at pH 7.0, the effect of the salt concentration of the binding buffer on the retention of antibodies was examined (Figure S3). As a result, elution of antibodies from HisMA20-EMA and HisMA20-EEMA columns was observed with PB at 50 and 100 mM salt concentrations. This may be due to the weakening of the electrostatic interaction between antibodies and the stationary phase as the salt concentration in the mobile phase increases. HisMA20-*t*BMA showed the strongest antibody retention and captured antibodies at all concentrations. Based on these results, we concluded that 10 mM PB at pH 7.0 is the optimal binding buffer. The salt concentration of the elution buffer was also further investigated at pH 3.0 (Figure S4), and it was concluded that 50 mM was the optimum concentration, which was applied for subsequent measurements. These results confirm that the HisMA20-EEMA column does not require any high salt concentration or specific additives for antibody retention at pH 7.0 and elution at pH 5.0.

### Gradient Elution of Antibodies

**Figure 3:**
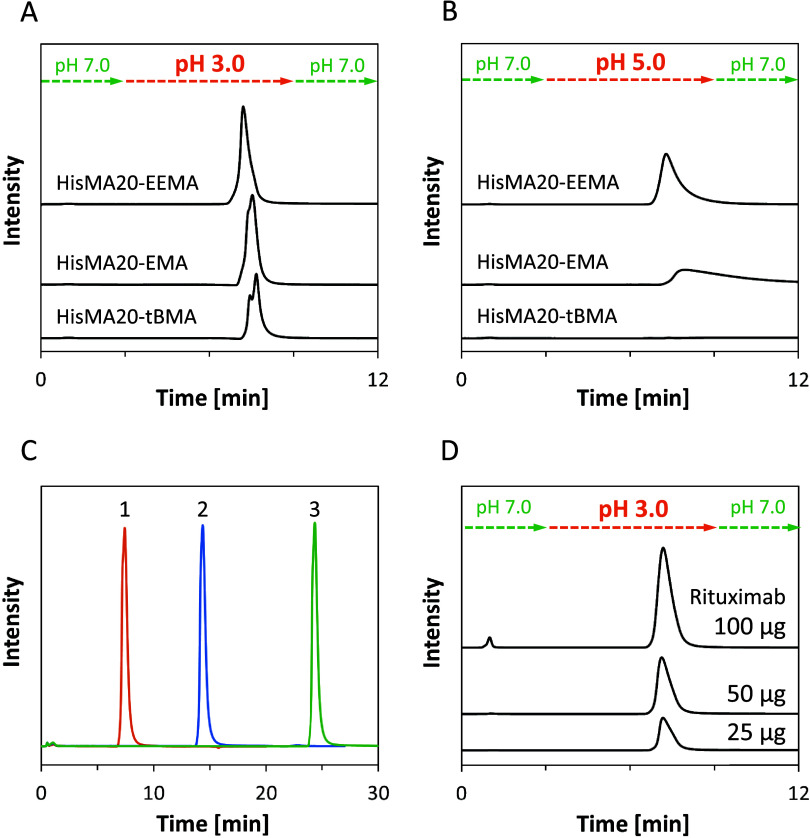
Chromatograms of gradient elution of rituximab using the HisMA20-EEMA column. Analytical conditions: flow rate: 0.2 mL/min; analyte: 4 mg/mL rituximab; injection volume: 2 μL; mobile phase: gradient elution starting with 10 mM PB at pH 7.0 for 3 min, followed by 50 mM CAB at (A) pH 3.0 (B) pH 5.0 for 6 min and finally 10 mM PB at pH 7.0 for 11 min. (C) Chromatograms of gradient elution of rituximab with different washing times with binding buffer (10 mM PB at pH 7.0), followed by elution buffer (50 mM CAB pH 3.0). Chromatograms 1, 2, and 3 correspond to 3, 10, and 20 min washing time with binding buffer, respectively. (D) Investigation of the effect of antibody concentration on the retention efficiency of the HisMA20-EEMA column (injection volume 10 μL).

Gradient elution of rituximab was conducted using binding and elution buffers with optimized salt concentrations. Following sample injection, the column was washed with binding buffer (10 mM PB at pH 7.0) for 3 min, after which the retained antibody was eluted from the column with elution buffer (50 mM CAB at pH 3.0 or 5.0) (Figure 3A,B). Comparison of results using 50 mM CAB at pH 5.0 as elution buffer (Figure 3B) shows a sharpness of the eluted peaks of antibodies in the order of HisMA20-EEMA, HisMA20-EMA, and HisMA20-*t*BMA. The higher the hydrophobicity of the neutral monomer in the pH-responsive mixed-charge polymer, the stronger the hydrophobic interaction with the antibody, making elution more difficult. The HisMA20-EEMA column eluted antibodies completely in about 2 min with pH 5.0 CAB buffer. Antibody recovery rates for each measurement are summarized in Figure S5. Notably, the HisMA20-EEMA column achieved an extremely high recovery (95.0 ± 1.4%) with pH 5.0 CAB as elution buffer. It was also confirmed that the antibodies captured on the HisMA20-EEMA column were not eluted by prolonged washing with binding buffer (10 mM PB at pH 7.0) (Figure 3C). The captured antibodies did not elute during 20 min wash with binding buffer, and eluted from the column after the eluent was switched to elution buffer (50 mM CAB pH 3.0). These results indicate that the retention of antibodies by the protein A mimetic column is due to a specific interaction between antibodies and the surface of the polymer modified resin under neutral conditions and not of nonspecific natures, such as the size exclusion effect of the resin.

Retention capacity tests were also performed using the HisMA20-EEMA column (Figure 3D). When 100 μg (10 μL of 10 mg/mL solution) of rituximab was injected into the column packed with 166 μL of the HisMA20-EEMA resin, a slight peak was observed at the washing step. The recovery was over 97% regardless of the amount of injected antibody. To calculate the maximum binding capacity, 100 μg rituximab was injected with binding buffer until the eluted antibody amount was saturated, after which the retained antibodies were eluted in one step by switching the mobile phase to pH 3 CAB (Figure S6). From the peak area and column volume, the binding capacity was calculated to be 5.51 mg/mL.

### Purification of mAbs from Mixed Samples

**Figure 4:**
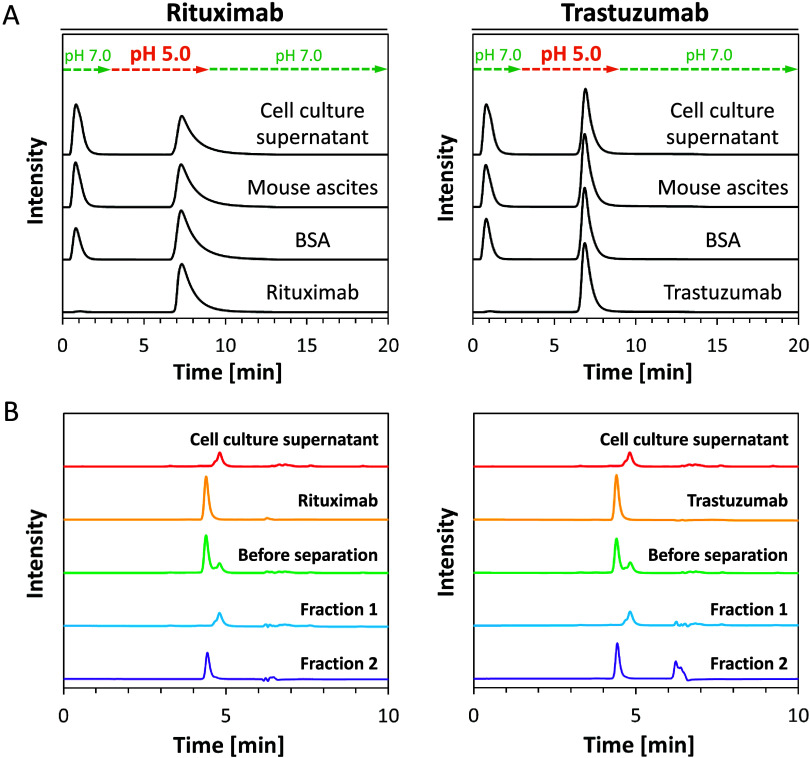
Purification of mAbs from mixtures with impurities using pH 5.0 elution buffer and SEC analysis of the fractions eluted from the HisMA20-EEMA column. (A) Chromatograms of purification of antibodies (2 mg/mL rituximab or trastuzumab) from mixtures with BSA, mouse ascites fluid, and hybridoma cell culture supernatant. (B) SEC analysis of fractions from the chromatograms of rituximab or trastuzumab in hybridoma cell culture supernatant. Fraction 1:0.5 to 1.5 min; fraction 2:7.0 to 8.0 min (rituximab), 6.6 to 7.6 min (trastuzumab) in the chromatograms (A).

**Figure 5:**
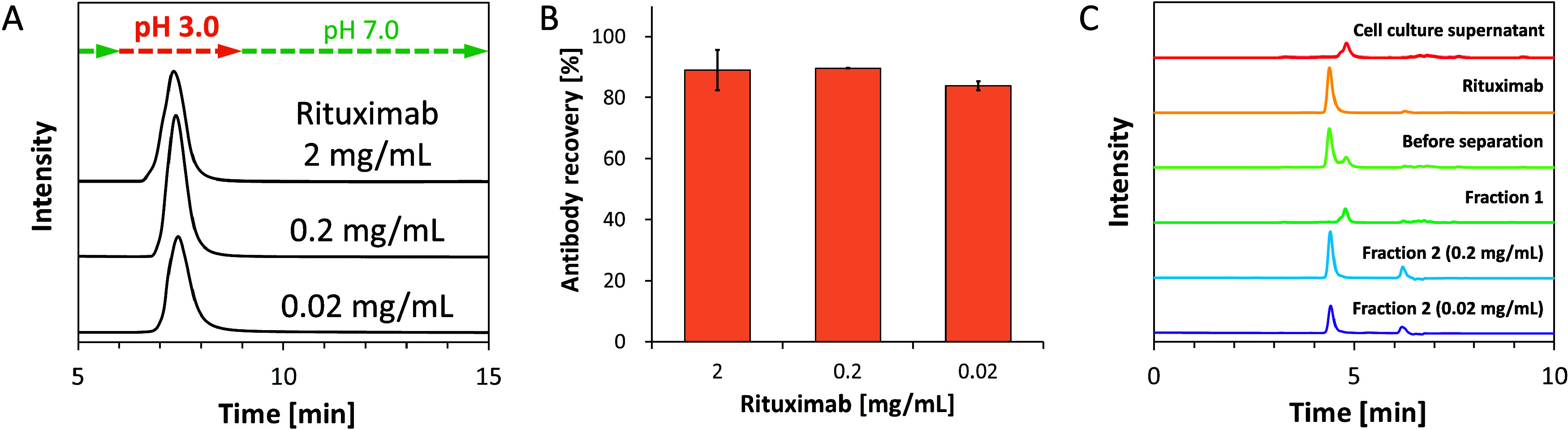
Purification of rituximab (2, 0.2, 0.02 mg/mL) in hybridoma cell culture supernatant. (A) Chromatograms of purification of rituximab (2, 0.2, 0.02 mg/mL) from hybridoma cell culture supernatant. Chromatograms of flow through (0–8 min) were removed to highlight the peaks of eluted rituximab. (B) Recovery of rituximab (2, 0.2, 0.02 mg/mL) from cell culture supernatant. Data represents mean values ± SD (*n* = 3). (C) SEC analysis of fractions from the chromatograms of rituximab in supernatant. Fraction 1:0.5 to 1.5 min; fraction 2:7.0 to 8.0 min in the chromatograms (A).

Purification of mAbs (rituximab, trastuzumab) from mixtures with BSA, mouse ascites fluid, and hybridoma cell culture supernatant was performed using the HisMA20-EEMA column with 50 mM CAB at pH 5.0 as elution buffer (Figure 4A). Antibody recovery rates from each mixed sample are summarized in Table S1. Both rituximab and trastuzumab were purified from BSA, mouse ascites fluid, and hybridoma cell culture supernatant with high recovery rates of 80.3–112.2% (Table S1). Eluted fractions were aliquoted, and the purity of purified antibodies was evaluated by SEC analysis; the results of SEC analysis of mAbs purified from hybridoma cell culture supernatant are shown in Figure 4B. Standard samples of mAbs (rituximab: 145 kDa and trastuzumab: 146 kDa)^49^ and hybridoma cell culture supernatant eluted at 4.4 and 4.8 min, respectively. In the SEC chromatograms of fraction 1 eluted with binding buffer, peaks derived from the hybridoma cell culture supernatant, but no mAb peak were detected. This indicates that impurities in the cell culture supernatant were eluted with the binding buffer, while the antibody was retained in the column. The SEC chromatograms of fraction 2 eluted with elution buffer showed a single peak of mAbs without any contaminants. The peak appearing at 6.0–6.5 min in fraction 2 from the purification of trastuzumab was derived from the elution buffer used for purification (50 mM CAB at pH 5.0) (Figure S7). No broadening or shoulders were observed in the peaks of fraction 2, and the peak shape matched that of the standard samples of antibodies, indicating that the purified antibody was not denatured or aggregated. These results confirm that the HisMA20-EEMA column allows purification of mAbs from hybridoma cell culture supernatant with very high efficiency. Antibody purification with 50 mM CAB at pH 3.0 as elution buffer was also performed by the HisMA20-EEMA column, and SEC analysis confirmed that antibodies purified from cell culture supernatant were not denatured (Figure S8). It was purified with high recovery rates (82.4–102.0%) similar to that with pH 5 (Table S1). SEC analysis of mAbs purified from mixtures with BSA and mouse ascites fluid was performed similarly, and excellent antibody purity was confirmed in all measurements (Figure S9). Furthermore, purification from cell culture supernatant containing rituximab at lower concentrations was performed for application to real samples (Figure 5A). To equalize the amount of antibody injected at one time, 10, 100, and 1000 μL of hybridoma cell culture supernatant containing rituximab at concentrations of 2, 0.2, and 0.02 mg/mL, respectively, was injected into the HisMA20-EEMA column. Antibody recoveries of 89, 90, and 84% were achieved from cell culture supernatant containing antibodies at concentrations of 2, 0.2, and 0.02 mg/mL, respectively (Figure 5B). The results of the SEC analysis indicate that the antibodies recovered from the low concentration samples were not denatured (Figure 5C). These results demonstrated that HisMA20-EEMA is also capable of capturing antibodies in low concentration samples.

### Reusability and Stability Test

**Figure 6:**
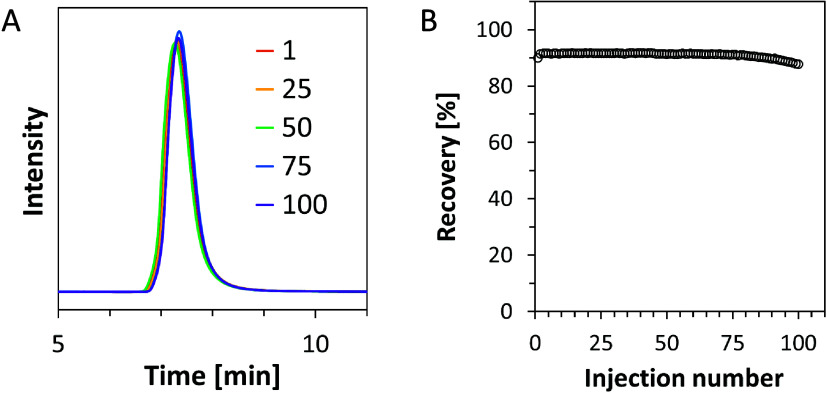
Effect of continuous purification on antibody recovery by the HisMA20-EEMA column. (A) Overlaid chromatograms of representative injections of rituximab (2 mg/mL) in hybridoma cell culture supernatant. Chromatograms outside the 5–11 min range were removed to highlight overlapping antibody peaks. (B) Summary of antibody recovery calculated from peak areas.

Reproducible antibody recovery performance in repeated purification operations is important for practical industrial applications. To investigate the reusability of the HisMA20-EEMA column, the purification of rituximab (2 mg/mL) from hybridoma cell culture supernatant with CAB at pH 3.0 as elution buffer was repeated continuously for 100 cycles. The chromatograms of representative injections and antibody recovery rates for each measurement are shown in Figure 6. The HisMA20-EEMA column reproducibly purified the antibody and maintained high recovery (90 ± 1.6%) even after 100 times purification. The pH 3 eluent and continuous injection of impurities did not cause any performance degradation, indicating that the HisMA20-EEMA column is highly reusable. Long-term stability of the column is also important for practical use. Figure S10 shows the chromatograms of the gradient elution of rituximab before and after six months of use. Retention time and number of theoretical plates after six months of use were 98.9 and 81.8%, compared with those before six months of use. These results indicate that the HisMA20-EEMA column has sufficient long-term stability.

## Conclusions

In this study, IgG-affinity polymers were successfully prepared by mimicking IgG-binding peptides with pH-responsive mixed-charge polymers. The column packing material modified with protein A mimetic polymers was shown to be able to switch the retention and elution behavior of antibodies by a simple manipulation of changing the pH of the mobile phase from neutral to acidic, similar to protein A affinity chromatography. The HisMA20-EEMA column successfully purified rituximab and trastuzumab from mixtures containing complex contaminants such as BSA, mouse ascites fluid, and hybridoma cell culture supernatant. Furthermore, the HisMA20-EEMA column showed reproducible antibody recovery (90 ± 1.6%) even after 100 consecutive purifications.

The HisMA20-EEMA column offers significant advantages over conventional antibody purification methods using affinity columns, including lower cost and higher durability due to the use of synthetic polymers, no leakage of affinity ligands to the purified antibody, and avoidance of harsh and potentially damaging antibody elution conditions. This concept of reproducing the function of affinity ligands by mimicking the amino acid sequence and structure of key amino acids with synthetic polymers could be applied to the development of inexpensive and durable purification methods for other target proteins.

## Supporting Information Available

Synthesis of HisMA; modification of V-501; absorbance of ninhydrin reaction solutions; SEM images; effect of salt concentration; antibody recoveries; maximum binding capacity; SEC analysis; stability test, and NMR spectra (PDF)
