# Supplementary material for: IgG-Binding Peptidomimetic Mixed-Charge Polymer-Modified Resins for Chromatographic Purification of Antibodies
Source: ACS Appl Mater Interfaces. 2024 Nov 26;16(49):67468–76. doi: 10.1021/acsami.4c16861 (PMC11647763; doi:10.1021/acsami.4c16861)

Supporting Information

IgG-Binding Peptidomimetic Mixed-Charge Polymer-Modified Resins for Chromatographic Purification of Antibodies

Koichi Deura[1], Akihiro Sakama[1], Yasuhiro Moriwaki[2], Daniel Citterio[1], and Yuki Hiruta[1]*

[1] Department of Applied Chemistry, Faculty of Science and Technology, Keio University, 3-14-1 Hiyoshi, Kohoku-ku, Yokohama, Kanagawa 223-8522, Japan

[2] Division of Basic Biological Sciences, Faculty of Pharmacy, Keio University, 1-5-30 Shibakoen Minato-ku, Tokyo 105-8512, Japan

*To whom correspondence should be addressed.

Email: hiruta@applc.keio.ac.jp

Table of Contents:

Scheme S1. Synthesis of the histidine-mimicking monomer HisMA

Scheme S2. Modification of V-501 on the surface of TOYOPEARL AF-Amino-650M resins

Figure S1. Absorbance of ninhydrin with TOYOPERAL and V-501-grafted TOYOPEARL

Figure S2. SEM images of TOYOPEARL, and V-501 and polymer-modified TOYOPEARL

Figure S3. The effect of salt concentration of binding buffer

Figure S4. The effect of salt concentration of elution buffer

Figure S5. Recovery of rituximab in gradient elution of rituximab

Figure S6. Maximum binding capacity test of the HisMA20-EEMA column

Figure S7. SEC analysis of fraction 2 from the purification of trastuzumab in hybridoma cell culture supernatant and aliquoted elution buffer without injection of trastuzumab

Table S1. Recovery of monoclonal antibodies from mixtures with BSA, mouse ascites fluid, and hybridoma cell culture supernatant

Figure S8. Purification of monoclonal antibodies from mixture with impurities using pH 3.0 elution buffer and SEC analysis of the fractions eluted from the HisMA20-EEMA column

Figure S9. SEC analysis of fractions from the chromatograms of monoclonal antibodies in mixture with BSA or mouse ascites fluid

Figure S10. Stability test for the HisMA20-EEMA column with rituximab

^{1}H NMR spectra

Abbreviations

DMF: *N*,*N*-dimethylformamide

quant.: quantitative yield

Scheme S1. Synthesis of the histidine-mimicking monomer HisMA.

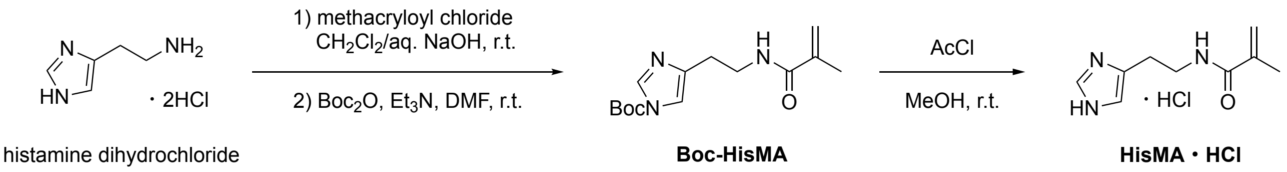

Synthesis of *N*-{2-[1-(*tert*-butoxycarbonyl)-1*H*-imidazol-4-yl]ethyl}methacrylamide (Boc-HisMA).

The following reaction was carried out under Ar. To a cooled (0 °C) stirred solution of histamine dihydrochloride (4.00 g, 21.5 mmol) in water (20 mL) were each added dropwise a solution of methacryloyl chloride (3.5 mL, 35 mmol) in CH_2Cl_2 (20 mL) and 4 M aqueous NaOH (20 mL, 80 mmol) with dropping funnels simultaneously over 30 min. The mixture was stirred at room temperature for 17 h, and methacryloyl chloride (0.43 mL, 4.4 mmol) and 5 M aqueous NaOH (0.90 mL, 5 mmol) were added at 0 °C. The mixture was stirred at room temperature for 2.5 h, and further methacryloyl chloride (0.43 mL, 4.4 mmol) and 5 M aqueous NaOH (0.90 mL, 4.5 mmol) were added at 0 °C. After being stirred at room temperature for 1.5 h, the mixture was concentrated under reduced pressure, and the residue was lyophilized and suspended in isopropyl alcohol (50 mL). The suspension was filtered, and the residue was washed well with isopropyl alcohol (50 mL). The combined filtrate and washings were concentrated under reduced pressure to give the crude HisMA (4.98 g) as a white solid, which was used in the next step without further purification.

The following reaction was carried out under Ar. To a cooled (0 °C) stirred solution of the crude HisMA (4.98 g) obtained above in DMF (43 mL) were added Boc_2O (5.3 mL, 24 mmol) and Et_3N (3.6 mL, 26 mmol). After being stirred at room temperature for 20 h, the mixture was quenched with water (120 mL) at 0 °C and extracted with ethyl acetate/hexane (1:1, 30 mL × 4). The combined extracts were washed with saturated brine (30 mL) and dried with anhydrous Na_2SO_4. The filtrate was concentrated under reduced pressure, and the residue was purified by flash column chromatography on silica gel (ethyl acetate/hexane, 3:1) to afford 580 mg of Boc-HisMA (10% for 2 steps) as a yellow oil: ^{1}H NMR (400 MHz, CD_3OD) δ 8.13 (s, 1H), 7.30 (s, 1H), 5.66 (s, 1H), 5.35 (brs, 1H), 3.49 (t, 2H, J = 7.0 Hz), 2.77 (t, 2H, J = 7.0 Hz), 1.92 (brs, 3H), 1.62 (s, 9H).

Synthesis of *N*-[2-(1*H*-imidazol-4-yl)ethyl]methacrylamide hydrochloride (HisMA·HCl).

To a cooled (0 °C) stirred solution of Boc-HisMA (233 mg, 0.835 mmol) in MeOH (8 mL) was added acetyl chloride (0.60 mL, 8.4 mmol). After being stirred at room temperature for 17 h, the mixture was concentrated under reduced pressure to afford 204 mg of HisMA·HCl (quant.) as a yellow oil: ^{1}H NMR (500 MHz, CD_3OD) δ 8.81 (s, 1H), 7.35 (s, 1H), 5.67 (s, 1H), 5.37 (brs, 1H), 3.55 (t, 2H, J = 6.7 Hz), 2.95 (t, 2H, J = 6.7 Hz), 1.91 (brs, 3H).

Scheme S2. Modification of V-501 on the surface of TOYOPEARL AF-Amino-650M resins.

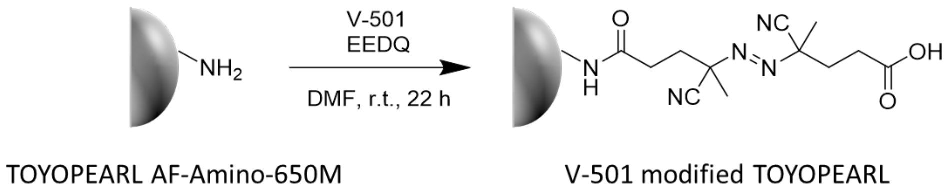

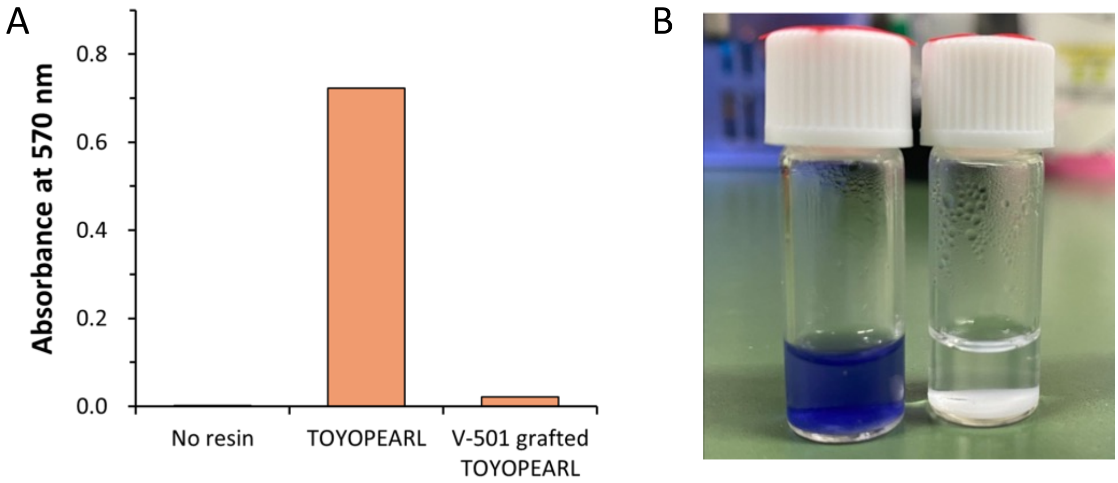

Figure S1. (A) Absorbance at 570 nm and (B) photographs of ninhydrin reaction solutions treated with TOYOPERAL (left) or V-501-grafted TOYOPEARL (right).

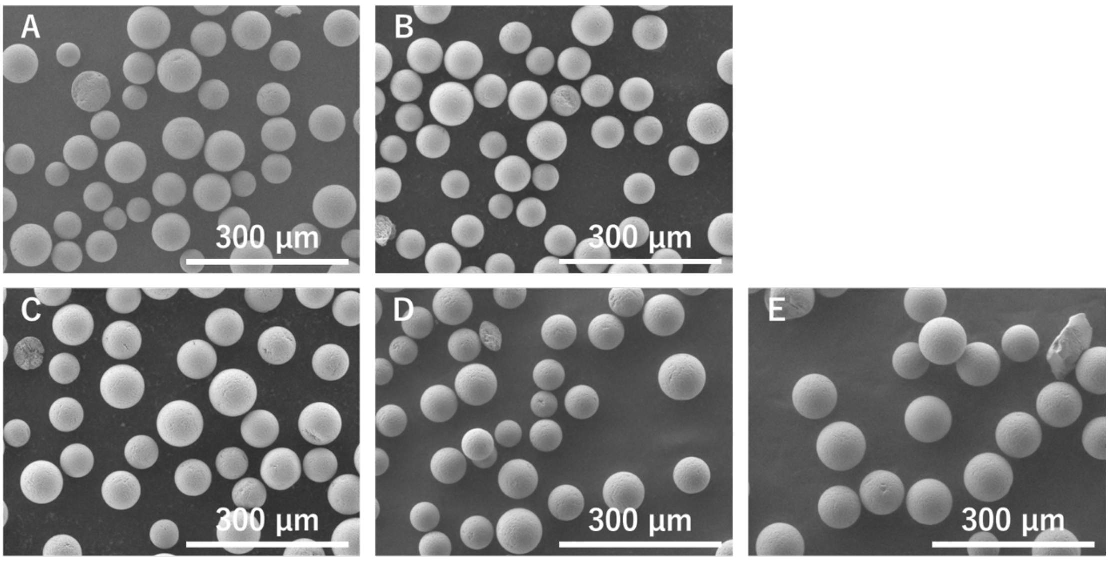

Figure S2. SEM images of (A) bare TOYOPEARL; (B) V-501-modified TOYOPEARL; (C) HisMA20-*t*BMA; (D) HisMA20-EMA; (E) HisMA20-EEMA.

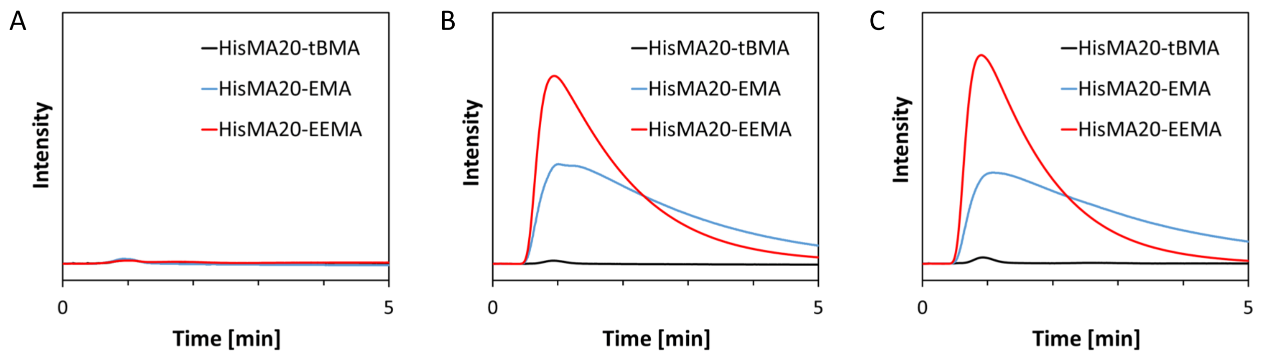

Figure S3. The effect of salt concentration of binding buffer. Analytical conditions: flow rate: 0.2 mL/min; analyte: 4 mg/mL rituximab; injection volume: 2 μL; mobile phase: (A) 10 mM PB at pH 7.0 (B) 50 mM PB at pH 7.0 (C) 100 mM PB at pH 7.0.

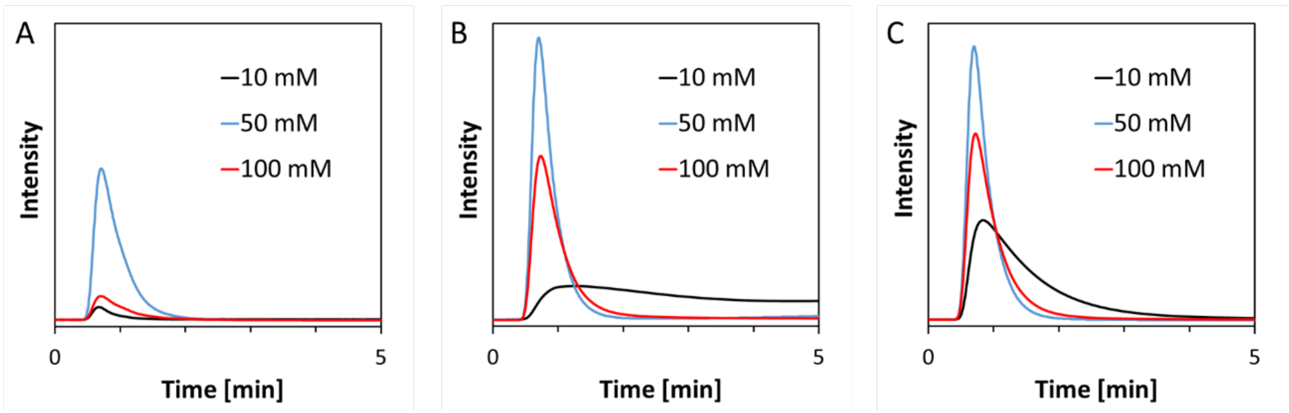

Figure S4. The effect of salt concentration of elution buffer in (A) HisMA20-*t*BMA (B) HisMA20-EMA (C) HisMA20-EEMA. Analytical conditions: flow rate: 0.2 mL/min; mobile phase: 10, 50, 100 mM CAB at pH 3.0; analyte: 4 mg/mL rituximab; injection volume: 2 μL.

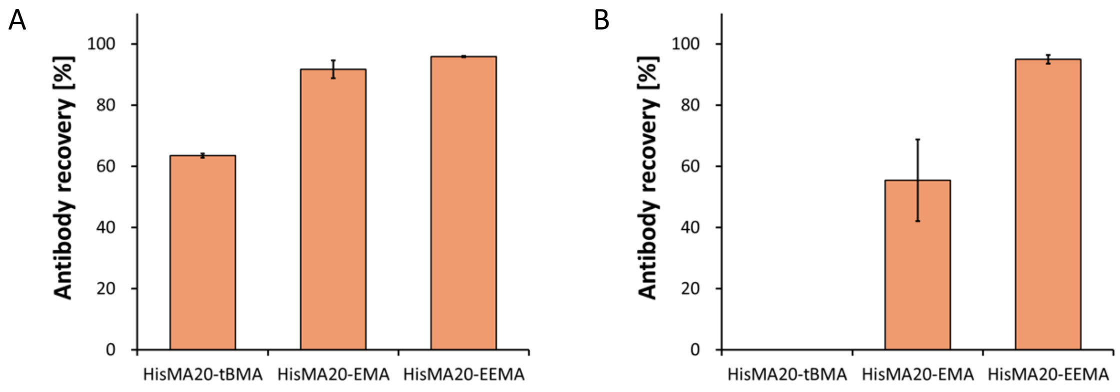

Figure S5. Recovery of rituximab in gradient elution using (A) 50 mM CAB at pH 3.0 (B) 50 mM CAB at pH 5.0 as elution buffer. Data represents mean values ± SD (n = 3).

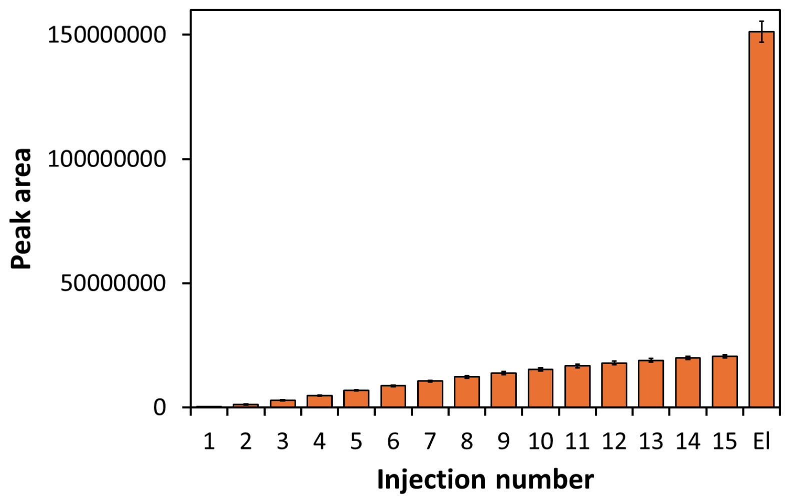

Figure S6. Maximum binding capacity test of the HisMA20-EEMA column. "El" represents the total amount of antibody bound to the column eluted at one time by 50 mM CAB at pH 3.0 after 15 injections of 100 µg rituximab (10 µL of 10 mg/mL solution). Data represents mean values ± SD (*n* = 3).

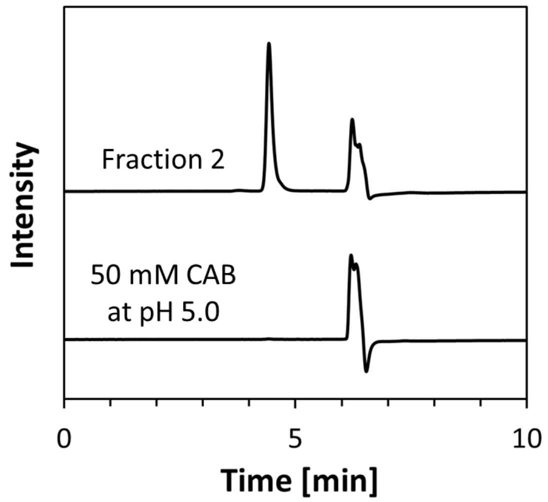

Figure S7. SEC analysis of fraction 2 from the chromatogram of trastuzumab in hybridoma cell culture supernatant and 50 mM CAB at pH 5.0, aliquoted elution buffer without injection of trastuzumab. Analytical conditions: flow rate: 0.35 mL/min; mobile phase: PB (0.2 M, pH 6.7, 0.05% NaN_3); injection volume: 20 µL.

Table S1. Recovery of rituximab and trastuzumab from mixtures with impurities in purification using the HisMA20-EEMA column. Data represents mean values ± SD (*n* = 3).

Impurity	Antibody recovery [%]			
	Rituximab		Trastuzumab	
	pH 3.0	pH 5.0	pH 3.0	pH 5.0
BSA	102.0 ± 3.7	112.2 ± 2.9	89.8 ± 5.7	89.7 ± 6.0
Mouse ascites fluid	96.0 ± 4.4	97.3 ± 3.2	96.6 ± 4.8	94.3 ± 6.5
Hybridoma cell culture supernatant	88.9 ± 6.5	84.3 ± 2.4	82.4 ± 4.4	80.3 ± 7.5

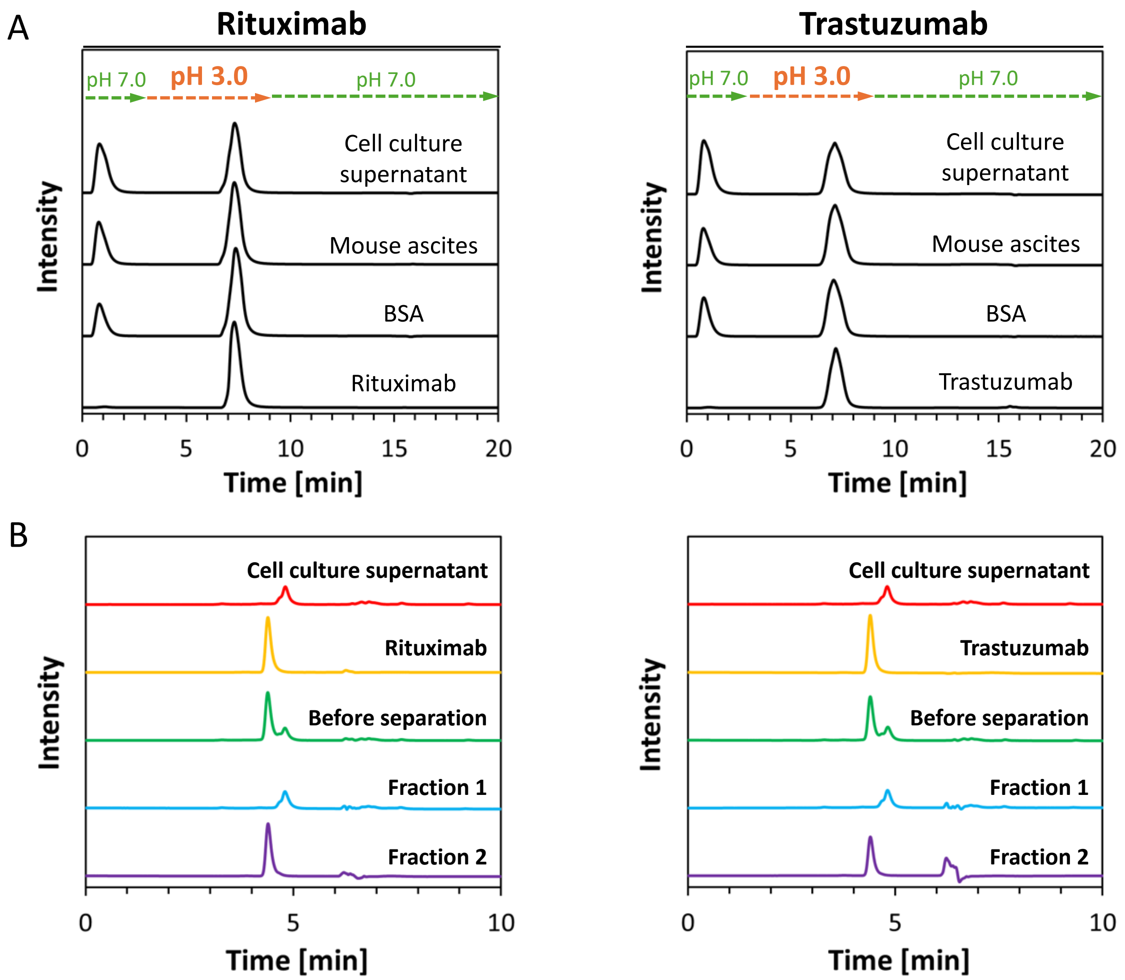

Figure S8. Purification of monoclonal antibodies from mixture with impurities using pH 3.0 elution buffer and SEC analysis of the fractions eluted from the HisMA20-EEMA column. (A) Chromatograms of purification of antibodies (2 mg/mL rituximab or trastuzumab) from mixtures with BSA, mouse ascites fluid, and hybridoma cell culture supernatant. (B) SEC analysis of fractions from the chromatograms of rituximab or trastuzumab in hybridoma cell culture supernatant. Fraction 1: 0.5 to 1.5 min; fraction 2: 6.8 to 7.8 min (rituximab), 6.7 to 7.7 min (trastuzumab) in the chromatograms (A). Analytical conditions: flow rate: 0.35 mL/min; mobile phase: PB (0.2 M, pH 6.7, 0.05% NaN_3); injection volume: 20 μL.

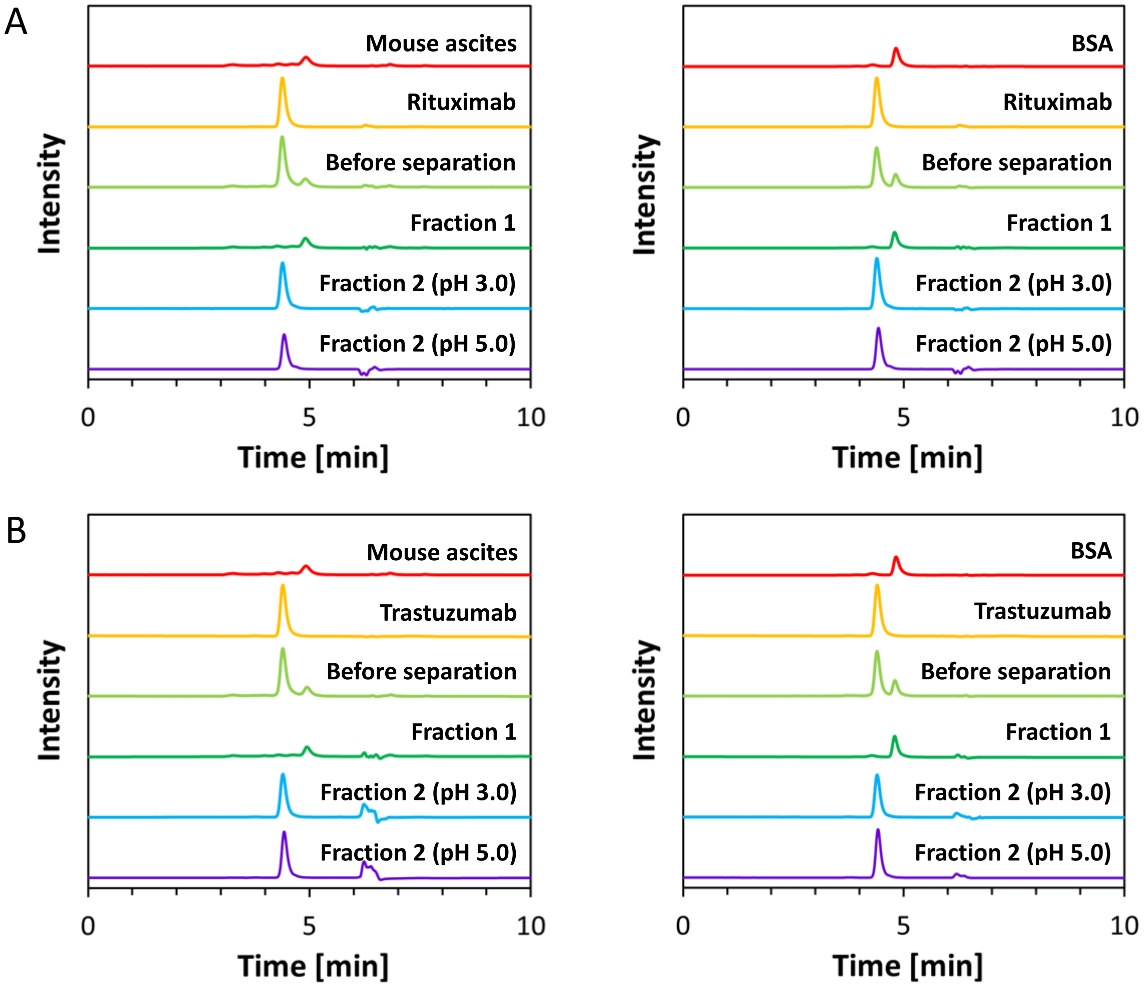

Figure S9. SEC analysis of fractions from the chromatograms of (A) rituximab or (B) trastuzumab in mixture with BSA or mouse ascites. Fraction 1 contains the impurities eluted with binding buffer and fraction 2 contains the monoclonal antibody eluted with elution buffer in each purification. Analytical conditions: flow rate: 0.35 mL/min; mobile phase: PB (0.2 M, pH 6.7, 0.05% NaN_3); injection volume: 20 µL.

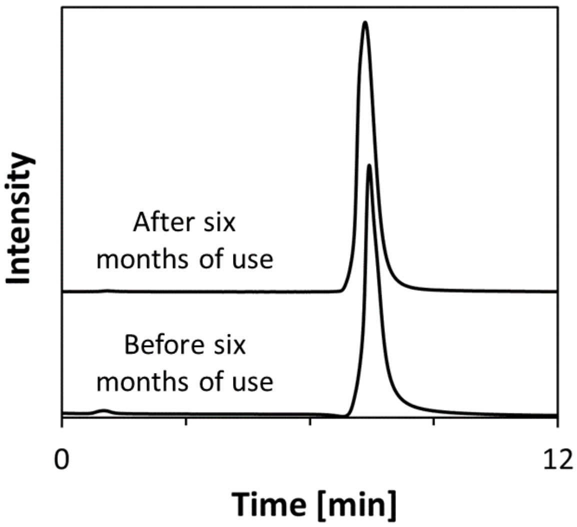

Figure S10. Stability test for the HisMA20-EEMA with rituximab. Analytical conditions: flow rate: 0.2 mL/min; analyte: 4 mg/mL rituximab; injection volume: 2 μL; mobile phase: gradient elution starting with 10 mM PB at pH 7.0 for 3 min, followed by 50 mM CAB at pH 3.0 for 6 min and finally 10 mM PB at pH 7.0 for 11 min.

^{1}H NMR spectra of Boc-HisMA.

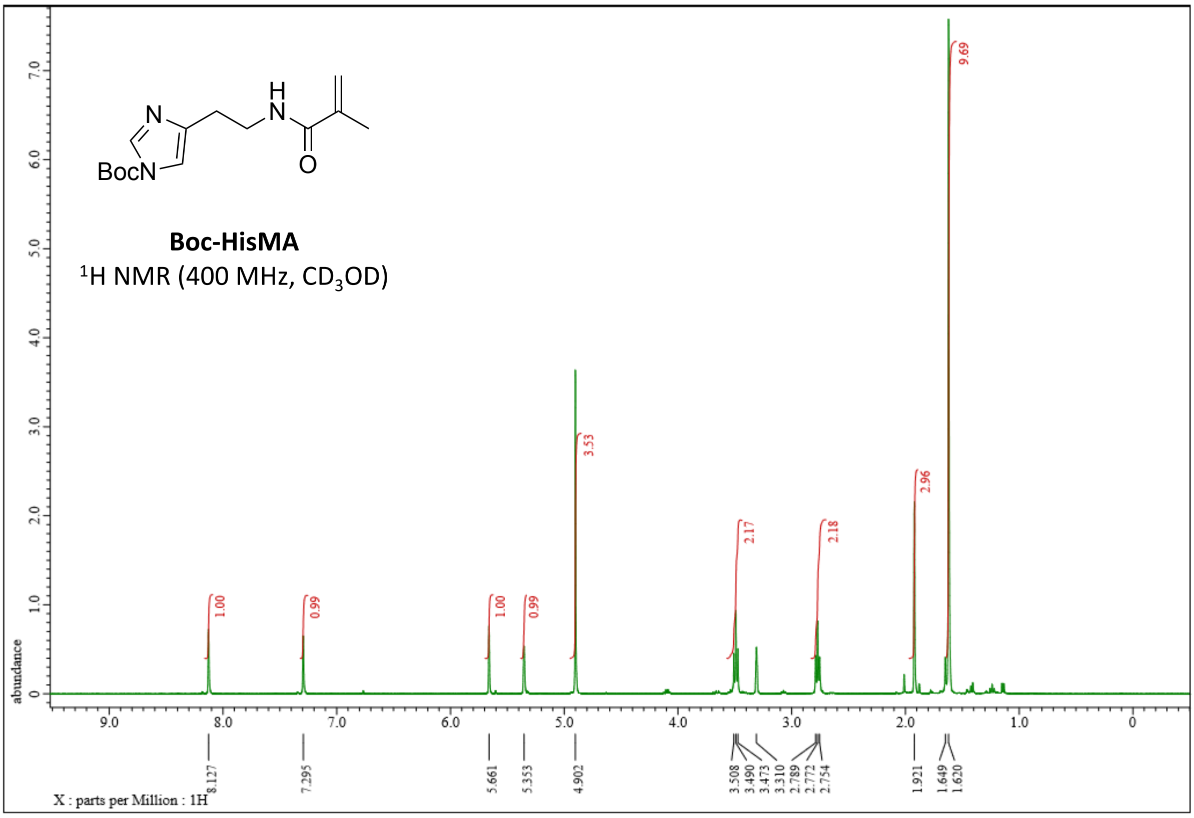

^{1}H NMR spectra of HisMA·HCl.

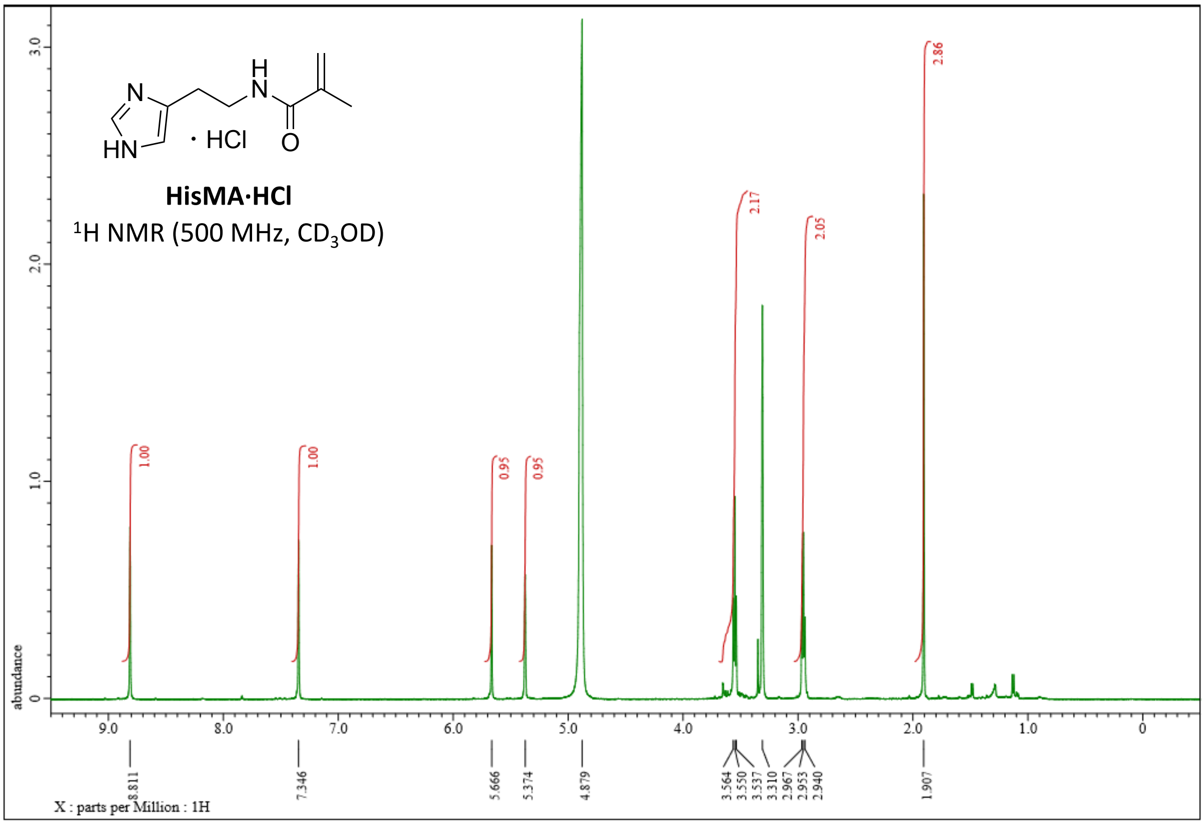

Supplement: Supplementary file 1 — am4c16861_si_001.pdf [file am4c16861_si_001.pdf]
